# IDO and TDO inhibitors in cancer immunotherapy: mechanisms, clinical development, and future directions

**DOI:** 10.3389/fphar.2025.1632446

**Published:** 2025-09-16

**Authors:** Raed M. Al-Zoubi, Mai Elaarag, Ahmad R. Al-Qudimat, Enas A. Al-Hurani, Zainab E. Fares, Ala’a Farhan, Sally R. Al-Zoubi, Abbas Khan, Abdelali Agouni, Mohanad Shkoor, Hiba Bawadi, Zain Z. Zakaria, Mazhar Al Zoubi, Khalid Alrumaihi

**Affiliations:** ^1^ Surgical Research Section, Department of Surgery, Hamad Medical Corporation, Doha, Qatar; ^2^ Department of Chemistry, Jordan University of Science and Technology, Irbid, Jordan; ^3^ Department of Biomedical Sciences, College of Health Sciences, QU-Health, Qatar University, Doha, Qatar; ^4^ Pharmaceutical Chemistry Department, Faculty of Pharmacy, Zarqa University, Zarqa, Jordan; ^5^ Department of Clinical Pharmacy, Jordan University of Science and Technology, Irbid, Jordan; ^6^ Department of Pharmaceutical Sciences, College of Pharmacy, QU Health, Qatar University, Doha, Qatar; ^7^ Department of Chemistry and Earth Sciences, Qatar University, Doha, Qatar; ^8^ Medical and Health Sciences Office, QU-Health, Qatar University, Doha, Qatar; ^9^ Department of Basic Medical Sciences, Faculty of Medicine, Yarmouk University, Irbid, Jordan; ^10^ Urology Division, Department of Surgery, Hamad Medical Corporation, Doha, Qatar; ^11^ Department of Urology, College of Medicine, Qatar University, Doha, Qatar

**Keywords:** IDO, TDO, cancer disease, enzyme inhibitor, mechanism, drug, safety, effectiveness IDO

## Abstract

Indoleamine-2,3-dioxygenase (IDO) and tryptophan-2,3-dioxygenase (TDO) inhibitors are promising avenues in cancer immunotherapy. These enzymes are key regulators in the kynurenine pathway. modulating immune responses and enabling tumor immune evasion. By targeting IDO and TDO. Therapeutic approaches aim to restore immune surveillance and enhance antitumor activity. This review examines the mechanisms of IDO/TDO in cancer etiology, their consequences in the tumor microenvironment, and the therapeutic development of inhibitors currently being studied. Among these, medications like Indoximod, Epacadostat, and Navoximod have shown promise in influencing the immune system and slowing tumor progression, while dual inhibitors like HTI-1090 try to address broader metabolic connections. Despite tremendous progress, obstacles like tumor heterogeneity, off-target consequences, and varying patient responses remain. The use of IDO/TDO inhibitors with conventional anticancer medications demonstrates their potential to reshape cancer treatment paradigms, contingent on further research to optimize efficacy and safety.

**Clinical Trial Registration:** https://clinicaltrials.gov/study/NCT03844438.

## Graphical Abstract

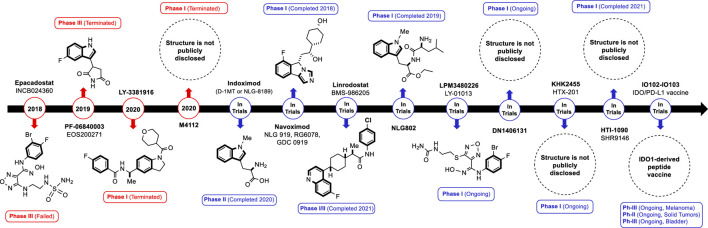

## 1 Introduction

Tryptophan is an essential amino acid that is important for multiple biological processes. It functions as a building block for proteins and is essential for the creation of several bioactive compounds necessary for immune system control, brain function and overall health. In addition, it is a precursor for the production of neurotransmitters such as serotonin, which affect mood regulation and cognitive function and melatonin, a hormone that is essential for controlling sleep cycles (Kanova and Kohout, 2021).

The metabolism of tryptophan made by enzymes such as indoleamine-2,3-dioxygenase (IDO) and tryptophan-2,3-dioxygenase (TDO) is an intriguing metabolic pathway with significant implications in a variety of physiological activities. IDO1 is found in multiple tissues and cells, including immune cells, playing an essential role in modulating immune responses and regulating inflammation and immune tolerance (Ye et al., 2019). IDO is an intracellular, monomeric, heme-containing enzyme that plays a huge role in catalyzing the first and rate-limiting step of the degradation of tryptophan. IDO1 degrades most of the tryptophan since it is diffusely expressed all over the human body in several organs such as the lung, spleen, liver, kidney, and brain. It catalyzes tryptophan by breaking the 2,3- double bond in tryptophan’s indole ring and a molecular oxygen (O2) is added into the open molecule, forming N-FK (Hornyak et al., 2018). IDO possesses two isoforms, IDO1 and IDO2, whose genes are successive and located on chromosome 8 (Najfeld et al., 1993). They display unique biochemical features and thus their activity depends on their place of origin or pathogenic site. IDO1 is highly expressed in peripheral lymph organs such as lymph nodes, spleen, and tonsils whereas IDO2 mRNA is expressed at much lower levels in the placenta and liver (Hornyak et al., 2018).

On the other hand, TDO functions in the liver, regulating systemic tryptophan levels. While tryptophan can also be metabolized to serotonin *via* tryptophan hydroxylase, most (90%–95%) is degraded by IDO and TDO into kynurenine (Rafice et al., 2009). The kynurenine pathway is activated by stress signaling hormones with two end products: quinolinic and picolinic acid. The TDO gene is localized on chromosome 4. TDO2 is another cytosolic heme-containing enzyme that participates in the first and rate-limiting step of tryptophan catabolization. TDO2 is a functional ortholog to IDO1, where they both participate in the same biochemical reaction (Bilir and Sarisozen, 2017). The homeostasis of these pathways is crucial for neurological integrity and immune regulation.

In cancer, IDO and TDO-mediated tryptophan catabolism contributes to immune suppression (detailed further in Mechanisms section) (Shadboorestan et al., 2023). By inhibiting these enzymes, therapeutic interventions aim to restore immune system activity and enhance antitumor immunity. Researchers want to boost the effectiveness of current cancer treatments by regaining the immune system’s capacity to identify and combat cancer cells by inhibiting IDO or TDO activity (Sun et al., 2025; Qiao et al., 2025; Dai et al., 2025). Several drugs targeting these enzymes are under investigation in clinical trials as adjuncts to traditional cancer treatments like chemotherapy, radiation, or immunotherapy (Zang and Dorff, 2025). The goal is to create a more hostile environment for cancer cells, making them more susceptible to the body’s immune response (Shadboorestan et al., 2023).

It has been demonstrated that inhibiting IDO and TDO may improve antitumor immune response (Yoshioka et al., 2022). Numerous medications that aim to inhibit these enzymes have been made and are now being researched as supplements to conventional cancer treatments. Due to the significant roles of IDO and TDO in cancer treatment, there is an increasing effort on developing inhibitors to target these enzymes for the treatment of cancer. Twelve IDO/TDO inhibitors are currently under clinical investigation, including selective IDO1 agents (epacadostat, BMS-986205, PF-06840003, navoximod, KHK2455, LY3381916), dual IDO1/TDO inhibitors (indoximod, HTI-1090, LPM-3480226, M4112), next-generation IDO1 inhibitor NLG-802, and the peptide vaccine combo IO102-IO103, with development ranging from Phase I to Phase II trials across various solid tumors.

IDO and TDO are important enzymes in the kynurenine pathway, which is highly controlled by many factors. The downstream effects of targeting these enzymes are complex due to dynamic interactions and feedback loops within the kynurenine pathway. Drug development depends on the understanding of this complex regulation of the kynurenine pathway in different tissues and diseases (Badawy et al., 2023). In addition, by modifying tryptophan metabolism and producing immunosuppressive metabolites, IDO and TDO contribute to immunosuppression. The exact mechanisms by which these enzymes influence T cells and dendritic cells, two critical immune cell types, are complex and highly context-dependent. It is crucial to identify the precise immunomodulatory pathways involved and how they affect different microenvironments (Dai et al., 2025; Xu et al., 2025).

Additionally, tumor microenvironments are heterogeneous, exhibiting differing concentrations of nutrients, oxygen, and immune cell infiltration. IDO/TDO expressions can be altered by these microenvironment factors for the development of targeted therapeutics, comprehending how the dynamic changes in the tumor microenvironment affect immune responses and the effectiveness of IDO/TDO inhibitors (Rohrig et al., 2015). Other additional metabolic pathways, such as those pertaining to amino acid metabolism and energy metabolism, cross paths with the kynurenine pathway. Disrupting IDO/TDO functions could have wider metabolic ramifications, impacting cellular redox status and energy balance. Striking a balance between therapeutic benefits and potential metabolic disruptions remains a challenge. Furthermore, variability in immune function, genetic background, and tumor characteristics contributes to differential patient responses. Certain intracellular signaling pathways, such as PI3K-Akt-mTOR, also modulate IDO/TDO activity, underscoring the need to understand the intricate regulatory networks governing these enzymes (Dehhaghi et al., 2019). Notably, IDO and TDO are expressed in non-tumor cells as well. To minimize off-target effects and ensure safety, it is crucial to understand the consequences of inhibiting these enzymes in healthy tissues (Théate et al., 2015).

To effectively address these complexities, a thorough understanding of the molecular and cellular mechanisms governing IDO and TDO in both health and disease is essential. Ongoing research is exploring optimal timing, dosing, and combination regimens to maximize the therapeutic potential of these inhibitors. This narrative review aims to provide an updated and comprehensive overview of these aspects.

## 2 Methods

This narrative review is based on a comprehensive literature search. Sources included peer-reviewed articles, clinical trial registries (e.g., ClinicalTrials.gov), and relevant conference abstracts. The primary databases searched were PubMed, Scopus, and Web of Science, using keywords such as “indoleamine 2,3-dioxygenase,” “tryptophan 2,3-dioxygenase,” “IDO1,” “IDO2”, “TDO2,” “cancer immunotherapy,” and “kynurenine pathway.” Additional references were identified through manual searches of cited literature in key articles. Studies were included if they focused on the biological roles, mechanisms of action, or clinical development of IDO and TDO inhibitors in cancer. No formal inclusion or exclusion criteria were applied, as is typical for narrative reviews, but an emphasis was placed on the most recent and clinically relevant data to provide an up-to-date and balanced perspective.

## 3 Role of IDO/TDO in cancer

### 3.1 IDO

A key mechanism connecting the immune system to the outside environment is tryptophan–kynurenine metabolism (Huang et al., 2020). The first enzyme in the pathway is called IDO, and it comes in two forms: IDO1 and IDO2. IDO1 and IDO2 have a greater affinity for tryptophan than TDO and can employ a variety of indole-derived chemicals as substrates. Immune tolerance is mostly determined by the kynurenine pathway (Stone and Williams, 2023). Antigen-presenting cells, including monocytes and dendritic cells (DCs), are the main source of constitutive IDO1 expression (Fallarino et al., 2002). When the T cell protein Cytotoxic-T-lymphocyte Antigen-4 (CTLA-4) binds to the (DCs’) B7 complex (CD80/86) on DCs, IDO1 expression is induced in the latter, leading to the production of anti-inflammatory compounds like kynurenic acid and 3-hydroxy-anthranilic acid (3-HAA). The use of abatacept or the antibody ipilimumab to block CTLA-4 is now a well-established anti-cancer medication, demonstrating the close relationship between IDO1 activation and autoimmune function as well as tumor formation (Belladonna et al., 2009).

While many studies have investigated IDO1 in the context of cancer, research on IDO2 remains relatively limited. High IDO1 expression has been linked to a bad prognosis for several malignancies (Weng et al., 2018). IDO1 has been suggested to be involved in creating immunological tolerance. The tumor suppressor Bin1, which is diminished in human malignancies, can block IDO1. Furthermore, a variety of cancer cells and antigen-presenting cells APCs demonstrate that IL-6, IFN-γ, TGF-β, CTLA-4 and programmed cell death protein 1 (PD-1) can all induce high levels of IDO1 (Munn and Mellor, 2013).

IDO1 contributes to immune suppression by promoting cancer cell proliferation, migration, and invasion. IDO1 first stimulates the production of immunosuppressive APCs by functioning as an intracellular signaling molecule without requiring enzymatic activity (Weng et al., 2018). By promoting carcinogenesis and aiding in the development of immunological checkpoints in cancer, IDO1 renders APCs tolerogenic. For instance, IDO1 produced in APCs might increase peripheral tolerance to tumor-associated antigens (TAAs) in tumor-draining lymph nodes, maintaining the activity of TAA-expressing malignant cells (Zhang et al., 2018). In addition, *via* the Kyn pathway, the activation of IDO1 in APCs indirectly controls nearby immune cells. While it stimulates CD4^+^ regulatory cells (iTreg) and myeloid-derived suppressor cells (MDSCs), it inhibits natural killer (NK) cells and CD8+T effector cells (Prendergast et al., 2018). None of them express IDO1. By speeding up the creation of Kyn and Trp consumption, activation of the Kyn pathway inhibits mammalian target of rapamycin (mTOR), which in turn suppresses CD8^+^ T cells and stimulates the formation of T cells and upgrades regulatory cells (Tregs). It also promotes aryl hydrocarbon receptor (AhR) and activates general control non-derepressible 2 (GCN2) (Munn and Mellor, 2013; Prendergast et al., 2018).

Moreover, NK cells and T-cell proliferation in the G1 phase can be inhibited by Trp intake, and certain toxic Kyn metabolites can cause T-cell death and specifically reduce T-helper 17 cells (Th17) (Zhang et al., 2018). Notably, IDO2-dependent B cell inflammatory states and IDO1-regulated Tregs may both play roles in cancer progression (Prendergast et al., 2018). Lastly, MDSCs can inhibit NK and T cells and promote the migration of cancer cells. Finally, MDSCs inhibit NK and T cells and facilitate tumor migration. IDO1 promotes IL-6, which enhances MDSC generation and migration into tumor tissues, creating an immunosuppressive microenvironment. Additionally, IDO1 increases IL-6 and downregulates IFN-γ to promote angiogenesis and tumor growth (Théate et al., 2015).

IDO1 contributes to tumor progression through immune suppression, tolerogenic APC induction, and MDSC-mediated effects, as detailed in the Mechanisms section (Munn and Mellor, 2013; Lewis-Ballester et al., 2016). Because IDO1 supports immune tolerance but also facilitates tumor progression, research has focused on developing IDO inhibitors. Recent studies show that IDO expression can be regulated by various endogenous molecules, suggesting that targeting these molecules to indirectly modulate IDO may also be effective. Some serine proteases, such as HtrA1, have been shown to increase IDO expression and may contribute to carcinogenesis (Clanchy et al., 2022; Zuo et al., 2014). In contrast, the bacterial quorum sensor PQS (*Pseudomonas* quinolone signal) inhibits both IDO1 and IDO2 expression (Og et al., 2022). Tumor cells may evade immune surveillance by increasing IDO expression in the tumor microenvironment. Selective IDO1 inhibitors like Epacadostat and Navoximod have been tested, but with limited success in some trials (Og et al., 2022).

### 3.2 TDO

Cancer tissues can express IDO1, TDO, or both. TDO has been shown to have immunomodulatory properties that promote tumor immune resistance and proliferation, therefore, TDO is considered a potential cancer treatment target due to the similar roles of TDO and IDO in regulating the Kyn pathway (Abdel-Magid, 2017). Some evidence suggests that cancer cells that overexpress TDO and activate AhR can avoid immune detection. Hepatic TDO predominantly metabolizes free plasma tryptophan to kynurenine and downstream metabolites. TDO is considered the principal regulator of plasma tryptophan concentrations due to its high capacity, despite its lower affinity compared to IDO (Cheong and Sun, 2018). Corticosteroids, commonly released in response to physical or psychological stress, stimulate TDO activity. TDO and IDO act as critical links between external stimuli and immune system modulation, with the kynurenine pathway also impacting the central nervous system (CNS). Quinolinic acid acts as an agonist at NMDA receptors, while kynurenic acid serves as an antagonist at the same sites (Stone and Williams, 2023).

TDO can be produced ectopically in a variety of malignancies, aiding immune evasion by generating immunosuppressive kynurenine even while IDO1 is suppressed. This functional redundancy implies that tumors may compensate for IDO1 inhibition by upregufiltration and medication penetration in the tumor microenvironment (Pilotte et al., 2012). Elevated TDO activity can also have an impact on systemic tryptophan depletion, indirectly influencing other organs and immune surveillance processes. This functional redundancy implies that tumors may compensate for IDO1 inhibition by upregulating TDO2, highlighting the need for dual inhibitors. TDO-driven kynurenine not only activates AhR in tumor cells and Tregs, but it also influences MDSC recruitment and function (Opitz et al., 2011). Hypoxia, local cytokine gradients (e.g., IL-6, IL-1β), and metabolic stress can all increase TDO expression, affecting immune infiltration and medication penetration in the tumor environment. Elevated TDO activity can also affect systemic tryptophan depletion, thereby affecting other organs and immune surveillance processes (Platten et al., 2019). Moreover, selective TDO inhibitors such as LM10 and 680C91, though still in preclinical stages, represent promising avenues to overcome TDO-driven immune resistance, and dual inhibitors like HTI-1090 aim to simultaneously target IDO1 and TDO to enhance therapeutic outcomes (Pilotte et al., 2012). Together, these aspects reinforce the need to target both IDO1 and TDO to overcome adaptive resistance mechanisms and improve treatment efficacy.

While most clinical efforts have focused on selective IDO1 inhibition, increasing evidence highlights the potential benefits of dual IDO1/TDO inhibitors for overcoming route redundancy. Agents such as HTI-1090 (SHR9146) and M4112 are among the few dual inhibitors that have entered clinical trials; however, their development is still in the early stages. Preclinical candidates like LM10 and 680C91 have shown the ability to inhibit both IDO1 and TDO, which supports increased antitumor immune responses by inhibiting compensatory TDO overexpression seen with IDO1 inhibition alone. Future research should prioritize the development and clinical translation of dual inhibitors, which may address adaptive resistance mechanisms and increase therapeutic efficacy across a wide range of tumor types.

## 4 Mechanism of action of IDO/TDO in cancer

**FIGURE 1:**
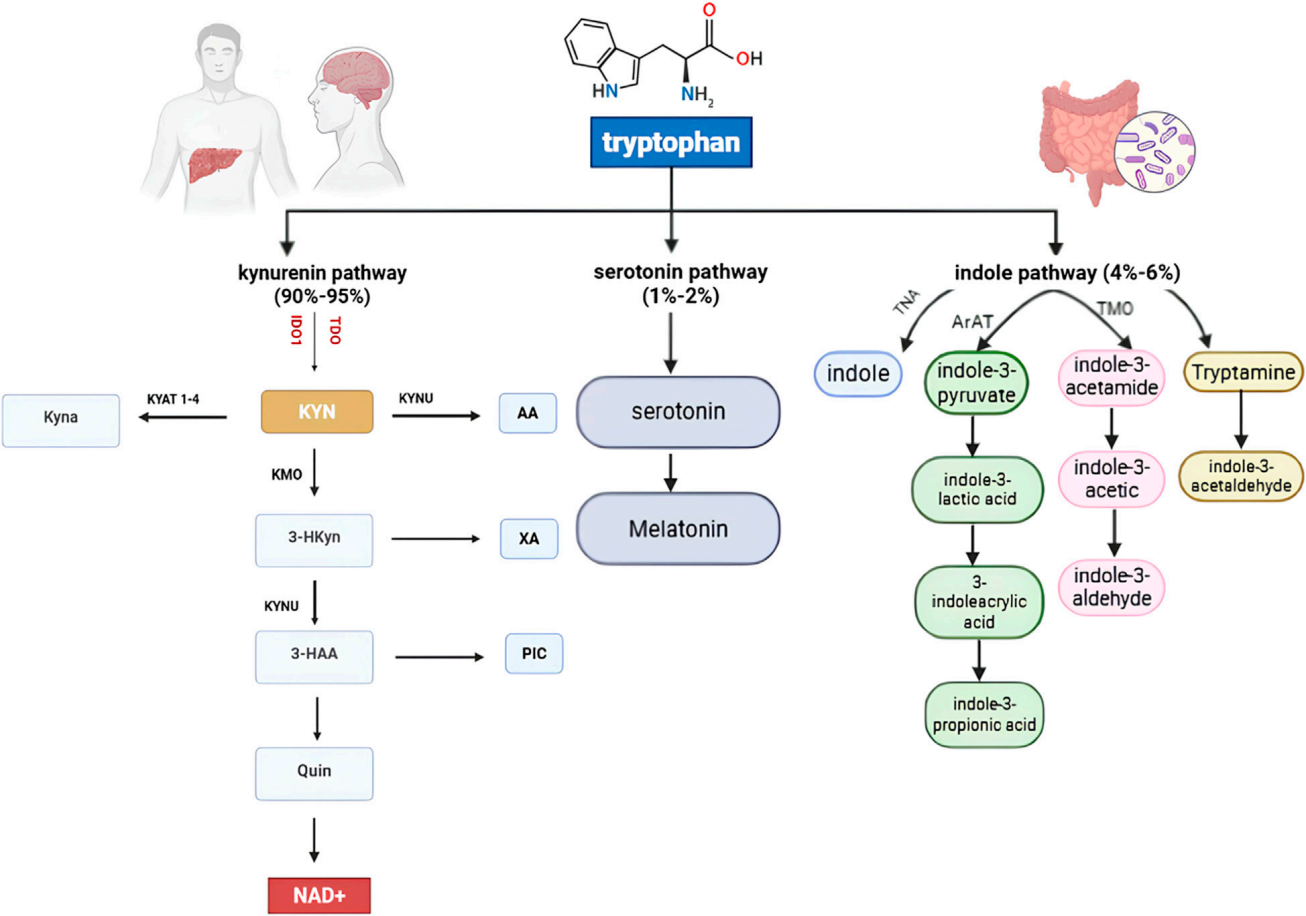
Tryptophan Metabolic Pathways: Tryptophan is primarily metabolized *via* the kynurenine pathway (90%–95%), producing NAD^+^ and various neuroactive metabolites. A minor fraction (1%–2%) enters the serotonin pathway to form serotonin and melatonin. The indole pathway (4%–6%), driven by gut microbiota, generates indole derivatives such as tryptamine and indole-3-acetic acid, influencing host–microbiome interactions. Key enzymes, including IDO1, TDO, KMO, and KYNU, regulate these interconnected metabolic routes.

The kynurenine (Kyn) pathway is a metabolic route in which tryptophan is degraded to kynurenine and other downstream metabolites. IDO and TDO enzymes play pivotal and similar roles in converting tryptophan to kynurenine, regulating the first and rate-limiting step of this pathway (Figure 1). The kynurenine pathway is essential for creating an immunosuppressive environment and contributes to immune privilege in certain sites. Consequently, IDO and TDO are directly or indirectly involved in various diseases, highlighting the need to fully understand their mechanisms to develop effective therapeutics (Ye et al., 2019).

One of the most widely investigated agents is indoximod, a tryptophan mimetic that reverses immunosuppression by modulating mTORC1 signaling and restoring T cell function (Fox et al., 2018). Tryptophan is an essential amino acid for protein synthesis and a serotonin precursor (Abdel-Magid, 2017). While a small fraction of tryptophan is converted to serotonin, the majority (90%–95%) is degraded through the kynurenine pathway, ultimately producing NAD+ as the final product (Ye et al., 2019; Thackray et al., 2008).

### 4.1 IDO1

IDO1 is highly expressed in various immune cells, fibroblasts, and tumor cells (Ye et al., 2019). It has lower substrate specificity and can bind to multiple substrates, including L-tryptophan, D-tryptophan, tryptamine, 5-hydroxytryptophan, and serotonin, whereas IDO2 and TDO have higher specificity. Beyond its enzymatic function, IDO1 also acts as a signaling molecule, influencing immune cell phenotypes toward immunoregulatory states (Albini et al., 2017; Mammoli et al., 2020). Inducibility is one of its key features; IDO1 is strongly induced by IFN-γ and other inflammatory cytokines such as TNF-α, IL-6, and IL-10 (Albini et al., 2017; Mammoli et al., 2020). The presence of two immunoreceptor tyrosine-based inhibitory motifs (ITIM1 and ITIM2) in IDO1 allows it to engage with SHPs and PI3K, promoting its long-term expression in dendritic cells under certain conditions (Albini et al., 2017; Mammoli et al., 2020). Bin1 can downregulate IDO1 through STAT1 and NF-κB-dependent pathways; however, in many cancers, Bin1 is inactivated, supporting IDO1 overexpression (Abdel-Magid, 2017). Additionally, two phosphorylatable tyrosine residues (Y115 and Y253) further regulate IDO1’s stability and activity by providing docking sites for signaling molecules (Albini et al., 2017; Mammoli et al., 2020).

IDO1’s overexpression leads to tryptophan depletion and accumulation of kynurenine metabolites, promoting inflammation and immune suppression by inhibiting effector T cells and NK cells and enhancing Treg and MDSC populations (Hornyak et al., 2018). Kynurenine and its metabolites also activate AhR, further promoting immunosuppressive phenotypes (Ye et al., 2019; Bilir and Sarisozen, 2017). Additionally, IDO1-mediated tryptophan depletion activates GCN2 kinase, leading to inhibition of T cell proliferation and promoting Treg differentiation (Ye et al., 2019; Tang et al., 2021). IDO1 downregulates IFN-γ and upregulates IL-6, thereby promoting angiogenesis and facilitating tumor growth (Ye et al., 2019). Additionally, Trp starvation and increased IDO1 activity result in:

• mTORC1 - the target of rapamycin-inhibition, which stimulates T-cell apoptosis, inhibition of T-cell proliferation, and APC-mediated inflammation (Hornyak et al., 2018).
• Activation of GCN2 which directly changes DCs to tolerogenic APCs and stimulates tumor-immune inhibitory cytokines such as IL-10 and TGF- ***β*** that block the conversion of Tregs to proinflammatory Th17 cells and instead upgrade them to stimulate tolerance (Ye et al., 2019).
• Activation of AhR through IDO1’s intracellular signaling function which promotes Treg differentiation and drives the promotion of DCs and macrophages to an immunosuppressive phenotype (Ye et al., 2019).

IDO1 also indirectly influences non-IDO1-expressing cells through the kyn pathway metabolites and helps in building immune checkpoints in cancer cells (Ye et al., 2019). Mesenchymal stem cells (MSCs) that normally do not express IDO1, are also induced to express IDO1 due to inflammatory stimulation by IFN-***γ*** and TNF- **α** (Bilir and Sarisozen, 2017). All these factors work together to suppress immunity and promote tolerance.

### 4.2 IDO2

In 2007 Metz et al. (2007) and Ball et al. discovered IDO2- which is an evolutionary paralog to the then-named IDO enzyme. IDO2 is located downstream of the IDO1 gene on chromosome 8, proposing that they arose from gene duplication (Metz et al., 2007), IDO2 shows a higher substrate specificity but a much lower expression level than IDO1. Furthermore, IDO2 has a lower affinity for tryptophan than both IDO1 and TDO, implying that IDO2 may have a different natural substrate due to its low enzymatic activity (Bilir and Sarisozen, 2017). IDO2 mRNA is expressed in the human liver, colon, spleen, small intestine, placenta, thymus, lung, brain, and kidney (Metz et al., 2007). It is also expressed in DCs, MSCs and APCs. IDO2 is induced by IL-10, LPS, AhR, prostaglandin E2, and IFN-***γ*** - which strongly induces IDO1 whereas it is considered a weak inducer of IDO2 (Li et al., 2021). In a study conducted on mice (Platten et al., 2019), it was revealed that the strong induction of IDO2 by TCDD (2,3,7,8-tetrachlorodibenzo-p-dioxin)—an activator of AhR—was dependent on the presence of RelB (NF-κB subunit). This finding suggests that RelB is necessary for the development of macrophages and DCs producing IDO2 in the thymus (Li et al., 2007). On the other hand, IDO2 expression and catalytic function are significantly inhibited by salinomycin resulting in the proliferation of CD8 + T cells (Ebokaiwe et al., 2020). Due to being a relatively recently discovered enzyme, IDO2 is less extensively studied than the other tryptophan catabolizing enzymes. However, it has garnered more attention in recent years due to the many possibilities and implications it can provide us with + it has been revealed in many studies that IDO2 is upregulated in many tumors.

### 4.3 IDO2 and cancer

The role of IDO2 has been suggested in many cancers such as non-small cell carcinoma, pancreatic cancers, and cervical cancer. As a result, it is presumed that IDO2 contributes to cancer development, but this relation is still not very well-studied (Li et al., 2021). IDO2 expression and its functional role vary depending on the type of tumor. Like its paralog IDO1, IDO2 expression is upregulated in most studied cancers, although some cancers show decreased IDO2 activity. IDO2 contributes to the formation of an immunosuppressive microenvironment through the overexpression of IDO2**.** Overexpression of IDO2 in tumors is less common than the overexpression of IDO1. However, it has garnered more attention in recent years due to the many possibilities and implications it offers. Additionally, several studies have revealed that IDO2 is upregulated in many tumors. Nevler et al. (2019) conducted a study on both mutant Kras transgenic mice with an IDO2 deficiency and cancer patients to address the correlation between IDO2 and PDAC development. It has been revealed that the loss of IDO2 is related to the reduction of PDAC tumor development in female transgenic mice and patients - suggesting that IDO2’s involvement in tumor development may be susceptible to sexual dimorphism to some extent. IDO2 overexpression was ascribed to the reduction of the neutrophil to overall lymphocytes, T-cell, and B-cell ratios. Moreover, IDO2 is correlated with an increase in the expression of cytotoxic lymphocytes. In a study by Mandarano et al. (2020), IDO2 expression was observed for the first time concerning non-small cell lung cancer (NSCLC) and suggested be contributing to poor prognosis. IDO2 overexpression and PD-L1 downregulation were observed in NSCLC adenocarcinoma, additionally, it was also associated with an intratumoral or mixed localization of the TILs - suggesting a role for IDO2 as an immunomodulatory molecule. The overexpression of IDO2 also correlated with a high PD-L1 expression in squamous cell carcinoma.

IDO2 possesses a role as a regulator of cytokine signalling by using a lipopolysaccharide (LPS) -induced endotoxin shock model, Yamamoto et al. (2018) studied the role of IDO2 as a cytokine signalling regulator by studying the peritoneal macrophages and T cells of IDO2 KO mice compared with WT mice. When cultured with LPS, peritoneal macrophages from IDO2 KO mice exhibited a significant increase in the production of cytokines including IL-1 **α** , IL-6, IL-10, MCP-1, MIP-1a, MIP-1b, and regulated the stimulation of normal T cell expression. In addition, cytokine production in T cells was similar in both IDO2 KO mice and WT mice. Consequently, it has been revealed that IDO2 overexpression inhibits cytokine signaling by studying RAW cells transfected with a GFP-tagged mouse full-length IDO2 (RAW-IDO2). IDO2 overexpressing RAW cells presented a decrease in IL-6, STAT3, SOCS1, and SOCS2 expression. However, there was no observed difference in NF-KB and stat1 expression compared to (RAW-MOC) - an empty vector-.

IDO2 inhibits the proliferation of CD4^+^ T and CD8^+^ T cells. Qian et al. (2012) established that IDO2 inhibits the generation of CD^+^4 T and CD^+^8 T cells, this suppression could not be reversed by either L- or D- 1-MT. In addition, the effect of increasing the trp concentration did not reverse the effect of IDO2- mediated T-cell suppression, unlike its paralog -IDO1- whose T-cell suppression effect could be reversed by 1-MT and by adding tryptophan.

The induction of IDO2 by AhR activation occurs by culturing T-cells with DCs expressing IDO2 (and IDO1), an increase in CD4^+^, CD25^+^, and Foxp3 Tregs percentages was noticed. Trabanelli et al. (2014) proved that IDO2 stimulation induces the generation of Tregs due to AhR activation. In addition, Liu et al. (2020) hypothesized that by using siRNA technology to silence IDO2 in DCs would activate them to enhance the anti-tumor response and suppress tumor progression. Following treatment with TNF-α, the percentage of mature DCs significantly increased, suggesting that IDO2 silencing can increase the sensitivity of DCs to TNF-α which can lead to DC maturation. Furthermore, it has been revealed that IDO2 silencing in DCs can initiate a potent T-cell response. Based on these studies, IDO2 might have a role in suppressing T-cell function and reinforcing the immunosuppressive microenvironment.

### 4.4 TDO

TDO is a tetrameric enzyme requiring reduction of its heme prosthetic group from ferric (Fe^3+^) to ferrous (Fe^2+^) form to oxidize the indole ring of tryptophan (Li et al., 2007). Similar to IDO2, TDO exhibits high tissue specificity. TDO expression is negligible in most tissues except for the liver, where it supports about 90% of hepatic tryptophan metabolism (van Baren and Van den Eynde, 2015) Thus, TDO is selectively expressed in hepatocytes, largely restricting its systemic effects, although it is slightly expressed in the brain (Badawy, 2017).

TDO has higher substrate specificity than IDO, with a Km of approximately 190 µM for L-tryptophan, allowing it to catabolize tryptophan at concentrations above physiological levels (∼80 µM) Under normal conditions, TDO controls plasma tryptophan levels to maintain homeostasis (van Baren and Van den Eynde, 2015).

TDO is induced in several ways, IL-6 indirectly induces the expression of TDO -and IDO- by upregulating the ISX gene, thus promoting the malignancy of hepatocellular cancer cells, the immunosuppressive PGE2 also induces TDO. L-trp indirectly activates TDO by promoting the production of reactive oxygen species (ROSs) (Li et al., 2007).

The activity of TDO is regulated by several mechanisms, glucocorticoid regulates TDO by the *de novo* synthesis of apoenzymes, tryptophan increases and regulates the activity of TDO, cofactor activation by the heme prosthetic, and feedback inhibition by NAD(P)H (Badawy, 2017).

TDO contributes to tumor immune resistance through the accumulation of kynurenine pathway metabolites. These metabolites play both independent and cooperative roles in tumor growth and immune evasion. Due to its higher Km, TDO efficiently produces kynurenine and its derivatives. These derivatives affect tumor progression by inducing CD4^+^CD25+Foxp3+ Tregs, promoting PD-1 expression in CD8^+^ T cells, and driving effector T cell apoptosis.

Kynurenine and its downstream products act as AhR agonists, fostering immunosuppressive microenvironments. L-kynurenine is particularly toxic to lymphocytes and promotes Treg differentiation. L-hydroxykynurenine inhibits CD4^+^ T cells and stimulates Tregs. 3-hydroxyanthranilic acid modulates monocyte and lymphocyte function, induces effector T cell apoptosis, and promotes Treg generation. Quinolinic acid enhances cancer cell proliferation, induces T cell apoptosis, and contributes to multidrug resistance. Picolinic acid inhibits effector T cell proliferation (Prendergast et al., 2018; Badawy, 2017).

### 4.5 IDO/TDO binding pockets

IDO and TDO are heme-containing enzymes that catalyze the oxidation of tryptophan, playing critical roles in immune regulation and cancer progression. The binding pockets and active sites of these enzymes are central to their catalytic efficiency, substrate specificity, and regulation of enzymatic activity. In IDO, the binding pocket is located within a large, hydrophobic cavity that allows for conformational flexibility, enabling the enzyme to bind a variety of substrates and inhibitors (Lob et al., 2009). This flexibility, attributed to loop regions that adopt multiple conformations, contrasts with TDO’s more constrained binding pocket, which is narrower and deeper due to its tetrameric architecture, imposing stricter steric and electronic requirements for substrate binding (F et al., 2007). The active sites of both enzymes feature a heme iron coordinated by a histidine residue, which is essential for catalyzing the oxidative cleavage of tryptophan to produce N-formylkynurenine. The surrounding residues in the active site play key roles in substrate orientation and stabilization, with subtle differences in hydrogen bonding networks and electrostatic environments influencing their catalytic mechanisms (Pantouris et al., 2014; Sugimoto et al., 2006). These structural features make their binding pockets prime targets for therapeutic intervention.

**FIGURE 2:**
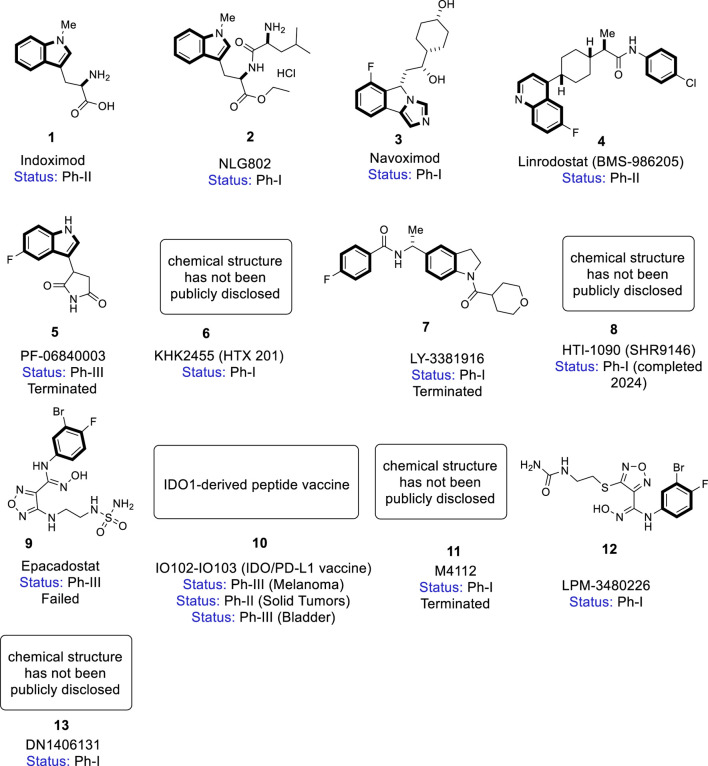
Chemical structures of IDO/TDO inhibitors in clinical development.

Current drug development efforts targeting IDO and TDO has gained significant momentum due to their roles in immune suppression and cancer progression, particularly through the kynurenine pathway. IDO inhibitors, such as epacadostat and navoximod (Figure 2), have been extensively studied in clinical trials for their potential to reverse immune evasion in cancers like melanoma and lung cancer (Beatty et al., 2017). These inhibitors exploit IDO’s flexible binding pocket, often mimicking the tryptophan substrate or interacting with the heme cofactor to block enzymatic activity (Long et al., 2019). However, the failure of epacadostat in the phase III ECHO-301 trial emphasizes the importance of robust biomarker-guided patient selection and rational combination strategies (Long et al., 2019; Zhou et al., 2025).

TDO inhibitors, though less advanced, are also being investigated for modulating systemic tryptophan levels and improving anti-tumor response. Compounds like LM10 and 680C91 have shown promise in preclinical studies by selectively binding to TDO’s rigid active site, offering a complementary approach to IDO inhibition (Opitz et al., 2011). Additionally, dual IDO/TDO inhibitors, such as RG70099 and HTI-1090, are being developed to overcome the redundancy IDO1 and TDO and provide broader pathway suppression (Labadie et al., 2019).

Moreover, integrating predictive biomarkers (e.g., baseline kynurenine/tryptophan ratios, tumor IDO/TDO expression profiles, and immune cell infiltration patterns) may help stratify patients and improve outcomes in future trials. Beyond oncology, these inhibitors are being explored for potential applications in neurodegenerative and autoimmune disorders linked to dysregulated tryptophan metabolism. Despite challenges, ongoing research into the structural and functional nuances of IDO and TDO binding pockets continues to drive innovation in drug design, with the goal of achieving greater selectivity, potency, and clinical success.

## 5 IDO/TDO inhibitors

### 5.1 Indoximod (1)

Indoximod (1**,** Figure 2, 1-methyl-D-tryptophan, D-1MT or NLG-8189**)** was developed by Newlink Genetics for stage IIb to stage IV melanoma. It contains indole and a [5,6]-fused heteroaromatic core (Fox et al., 2018). Initially investigated as a racemic compound (1-methyl-D, L-tryptophan), it showed anti-tumor activity in preclinical studies (Friberg et al., 2002). The L- and D-isomers show weak binding to IDO1, but the L-isomer is more active against IDO2, while the D-isomer acts primarily as a tryptophan mimetic, reversing mTORC1 inhibition induced by tryptophan depletion in human T_EFF_ cells (Prendergast et al., 2017; Prendergast et al., 2014).

In phase I trials, it was well-tolerated as single agent or in combination with chemotherapy in studies that established a dose of 1,200 mg/day for ongoing evaluation in multiple Phase II trials (Soliman et al., 2014; Soliman et al., 2016). Then indoximod entered phase II, it has been studied in several types of cancer (see Table 1 for detailed results) showed that the combination of indoximod with other therapies including cancer vaccines (Sipuleucel-T/Adenovirus-p53 transduced dendritic cell (DC) Vaccine), checkpoint inhibitors (pembrolizumab/nivolumab/Ipilimumab) and chemotherapy showed markedly enhanced the antitumor efficacy (Jha et al., 2017; Soliman et al., 2018). Due to the bioavailability limitations, a prodrug, NLG802 (**2,** Figure 2), was developed.

**TABLE 1:** Summary of IDO/TDO inhibitors.

Drugs/Pro-drugs/Compound no.	Commercial/abbreviated name	Status	Mechanism of action	Cancer type/type of cell lines	Type of medication/dose	Co-therapy	Results (efficacy and safety)	NCT number	References
Indoximod/1	D-1MT/NLG-8189	Phase I/II (Completed)	IDO1 activity inhibition; stimulates mTORC to modulate downstream transduction; promotes kynurenate formation	Melanoma Metastatic castration resistant prostate cancer (mCRPC) Metastatic Breast Cancer	1,200–1,600 mg BID	Ipilimumab Nivolumab DC vaccines	ORR: 36%–56% common AEs: fatigue, diarrhea, anemia	NCT02073123 NCT01560923 NCT01042535	(Zakharia et al., 2021) (Jha et al., 2017) (Soliman et al., 2018)
NLG802/2	—	Phase I/II (Completed)	ERα degradation, blocks estrogen signaling	HR+ metastatic breast cancer	Not yet established	CDK4/6 inhibitors (palbociclib, ribociclib, abemaciclib)	Preliminary tumor shrinkage common AEs: nausea, fatigue	NCT03164603	NewLink et al. (2020), Mautino et al. (2017)
Navoximod/3	NLG 919 GDC 0919	Phase I (Completed)	IDO1 pathway inhibition	Recurrent advanced solid tumors	50–1,000 mg BID	Atezolizumab	ORR: 9%–11% common AEs: fatigue, rash	NCT02048709 NCT02471846	(Nayak-Kapoor et al., 2018) (Jung et al., 2019)
Linrodostat/4	BMS-986205	Phase I/II (Completed)	Irreversible IDO1 inhibition	Selected solid tumors	100–200 mg QD	Nivolumab	ORR: 17%–37% common AEs: fatigue, nausea	NCT02658890	Siu et al. (2017), Tabernero et al. (2018), SITC (2017)
PF-06840003 (EOS200271)/5	—	Phase I (terminated)	Selective IDO1 inhibition	Malignant glioma	50–2000 mg BID	-	ORR: 0% well tolerated	NCT02764151	Reardon et al. (2020)
KHK2455/6	—	Phase I (Completed)	IDO1 apo-enzyme inhibition	Solid tumor	50–800 mg QD	mogamulizumab	ORR: 0% common AEs: nausea, fatigue	NCT02867007	Sahebjam et al. (2020a)
LY3381916/7	—	Phase I (Terminated)	Potent, selective IDO1 inhibition	Solid tumor	50–800 mg QD/BID	Anti-PD-L1 (LY3300054)	ORR: 0% common AEs: liver toxicity in TNBC cohort	NCT03343613	Kotecki et al. (2021)
HTI-1090 (SHR9146)/8	—	Phase I (Completed)	Dual IDO/TDO inhibition	Solid tumor	100–600 mg BID	Camrelizumab ± apatinib	ORR: 21%–33% acceptable safety	NCT03491631	Cheng et al. (2021)
Epacadostat/9	INCB024360	Phase II (Terminated)	Potent, selective IDO1 inhibition	Ovarian Melanoma	600-100 mg BID	Tamoxifen Pembrolizumab vs. placebo+ pembrolizumab	PFS ∼4–5 months modest efficacy common AEs: fatigue	NCT01685255 NCT02752074	(Kristeleit et al., 2017) (Long et al., 2019)
IO102-IO103/10	—	Phase I/II (Ongoing)	IDO and PD-L1 vaccine activation	Melanoma NSCLC	200 mg IV Q3W	pembrolizumab	ORR: 48%–80% common AEs: injection-site, irAEs	NCT03047928	Kjeldsen et al. (2021), Tucker (2023)
M4112/11	—	Phase I (terminated)	Dual IDO1/TDO inhibition	Advanced solid tumors	50–1,200 mg QD	–	ORR: 0%; failed to suppress Kyn; well tolerated	NCT03306420	Merck Serono SA, Synapse patsnap M-4112 (2025), Naing et al. (2020)
LPM3480226/12	LY-01013	Phase I (Ongoimg)	Dual IDO1/TDO inhibition	Advanced solid tumors	Dose not yet fully disclosed	—	Preliminary tumor shrinkage; acceptable safety	NCT05428774	Luye Pharma Group Ltd (2025), ClinicalTrials (2019a)
DN1406131/13	—	Phase I (ongoing)	Dual IDO1/TDO inhibition	Advanced solid tumors	First-in-human; dose-escalation ongoing	—	Preliminary data suggest acceptable safety	NCT05755436	Opitz et al. (2020), Clinical Trials (2019b), Peng et al. (2022)

Abbreviations: **AEs**, Adverse events; ORR, objective response rate; PFS, Progression-free survival; BID, twice daily; QD, once daily; DC, dendritic cell; ERα, estrogen receptor alpha; CDK4/6 , Cyclin-dependent kinase 4 and 6; PD-L1, Programmed death-ligand 1; PD-1, Programmed death-1; TNBC, Triple-negative breast cancer; NSCLC, Non-small cell lung cancer; IV, intravenous; Q3W , Every 3 weeks.

### 5.2 NLG802 (2)

NLG802 (**2,** Figure 2, ethyl Na-(L-leucyl)-1-methyl-D-tryptophanate) is an indoximod prodrug, designed to overcome the limited bioavailability of its parent compound. It improves systemic exposure and enhances immunomodulatory effects by ensuring higher plasma concentrations of indoximod (Kumar et al., 2020). Preclinical studies demonstrated superior pharmacokinetics, with increased oral bioavailability (>5-fold), higher Cmax, and greater AUC in animal models compared to equivalent doses of indoximod. It has completed a phase I clinical trial (NCT03164603), showing favorable absorption, rapid metabolism to active indoximod, and a favorable safety profile (NewLink et al., 2020; Mautino et al., 2017). Current phase 1/2 trials assess its safety, efficacy, and potential use in combination with other immunotherapies. While not yet FDA-approved, NLG802 represents a promising strategy to improve clinical outcomes through better pharmacokinetic performance and potentially broader patient benefit.

### 5.3 Navoximod (3)

Navoximod (**3**, Figure 2; also known as NLG919, RG6078, GDC-0919) is an orally active IDO1 inhibitor developed by NewLink Genetics. It contains a phenylimidazole core that enables it to inhibit IDO1 by binding to heme iron at the active site. Navoximod reduces kynurenine levels and restores T-cell function (Prendergast et al., 2018). In the clinical setting, Navoximod has been studied as monotherapy in patients with recurrent/advanced solid tumors. The study demonstrated that Navoximod **3** was generally well tolerated at doses up to 800 mg BID decreasing plasma kynurenine levels consistent with its half-life (Nayak-Kapoor et al., 2018). Another completed phase I clinical study of Navoximod combined with Atezolizumab to treat locally advanced or metastatic (Table 1). In combination with atezolizumab, navoximod demonstrated acceptable safety, but no clear evidence of additional benefit over atezolizumab alone was observed (Jung et al., 2019). Further research has revealed that absence of biomarker-guided patient selection may have contributed to limited clinical effect, stressing the need of incorporating kynurenine/tryptophan ratios and tumor IDO1 expression in future trials.

### 5.4 Linrodostat (4)

Linrodostat (4, Figure 2, also known as BMS-986205**)** is a potent, selective, irreversible oral IDO1 inhibitor developed by Bristol Myers Squibb. It structurally belongs to the class of 1-(4-arylcyclohex-1-yl) propanamides (Fraunhoffer et al., 2019). Linrodostat occupies the heme cofactor binding site to block IDO1 activation, preventing tumor immune evasion (Balog et al., 2021). Several phase I/II studies suggest that combining BMS-986205 with nivolumab is safe and could enhance response rates among patients with bladder and cervical cancers. The recommended dose for further study was 100 mg daily (Blocking IDO1 Helps Shrink Bladder and Tumors, 2018; Luke et al., 2019). Despite promising first results, further confirmatory data are needed to establish its efficacy across diverse tumor types, particularly given tumor heterogeneity and varying IDO1 expression in the tumor microenvironment.

### 5.5 PF-06840003 (5)

PF-06840003 **(5,** Figure 2, also known as EOS200271**)** is a tryptophan non-competitive, non-heme binding IDO1 inhibitor co-developed by Pfizer and iTeos. Its structure of is distinct from other IDO1 inhibitors. It inhibits IDO1 without directly coordinating to the heme iron atom (Crosignani et al., 2017).

PF-06840003 was dsigned to exhibit good pharmacokinetic properties, a longer half-life potentially allowing for single daily dosing, and CNS penetration for possible use against brain metastases (Crosignani et al., 2017). A phase I study A phase I study evaluated it as monotherapy in patients with recurrent malignant glioma but was prematurely terminated by the sponsor, and no further development for glioma was pursued (Table 1) (Reardon et al., 2020). The termination highlights the challenges of targeting IDO1 in CNS malignancies and the importance of integrating combination strategies or improved CNS-specific formulations in future designs.

### 5.6 KHK2455 (6)

KHK2455 **(6,** Figure 2, also known as HTX201 is a novel oral IDO1 inhibitor developed by Kyowa Kirin. It selectively inhibits inactive apoenzyme form of IDO1, providing longer and more potent activity compared to inhibitors that bind only to active holoenzyme (Sahebjam et al., 2020a).

It is currently in phase I clinical trials; it is being assessed in combination with mogamulizumab (an anti-CCR4 monoclonal antibody) in subjects with advanced solid tumors (Table 1). Results showed that KHK2455 was well-tolerated with tolerable toxicity at all doses tested, suppressed Kyn production in a dose-dependent and sustained manner, and demonstrated signals of antitumor activity (Dorsey et al., 2018). Future research should look into patient screening strategies and mechanistic biomarkers to maximize the efficacy of KHK2455, particularly in combination regimens targeting immunosuppressive cells.

### 5.7 LY3381916 (7)

LY3381916 **(7,** Figure 2) is an indoline derivative developed by Eli Lilly. It is a potent and highly selective IDO1 inhibitor, with an IC50 of 7 nM against IDO1 and >20 μM against TDO, underscoring its selectivity (Dorsey et al., 2018). It was studied in a phase I trial as monotherapy and in combination with PD-L1 inhibitor LY3300054 (table 1). Although safe to administer alone at up to 240 mg daily, the combination in triple-negative breast cancer was limited by grade 3 liver toxicity in 35.7% of patients, which prevented further escalation (Kotecki et al., 2021). These findings highlight the importance of careful safety monitoring and potential dose adjustments, particularly when used in combination with checkpoint inhibitors in sensitive patient populations.

### 5.8 HTI-1090 (8)

HTI-1090 (8, Figure 2, also known as SHR9146) is a highly potent dual inhibitor targeting both IDO1 and TDO, developed by Jiangsu Hengrui Medicine. It was designed to address functional redundancy in the kynurenine pathway by simultaneously blocking both enzymes. Additionally, it had favorable safety profiles and oral bioavailability in preclinical studies (Tu et al., 2019). Phase I studies as monotherapy or in combination therapy for patients with solid tumors showed promising anti-tumor activity with acceptable safety in patients with advanced solid tumors. Further study is needed to validate the efficacy and safety as shown in Table 1 (Cheng et al., 2021). However, more research is needed to find out how these strategies work over the long term and which groups of patients are most likely to benefit from them.

### 5.9 Epacadostat (9)

Epacadostat (**9,** Figure 2, also known as INCB024360) a highly potent, selective, orally available IDO1 inhibitor developed by Incyte Corporation. It has an IC_50_ of 10 nM and minimal activity against IDO2 and TDO (Liu et al., 2010). It displays immunomodulating and antineoplastic activities by inhibiting IDO1, leading to decreased kynurenine levels and restoring proliferation and activation of immune cells suppressed in many cancers (Dhiman et al., 2017). Over 50 clinical trials evaluated epacadostat as monotherapy or in combination with checkpoint inhibitors such as ipilimumab, nivolumab, durvalumab, and pembrolizumab in multiple malignancies including melanoma, NSCLC, SCCHN, and ovarian cancer (Beatty et al.; Hellmann et al., 2020; Doi et al., 2021). In early-phase trials, epacadostat was generally well tolerated, normalized plasma kynurenine levels, and showed maximal IDO1 inhibition at doses >100 mg BID (Beatty et al.; Komiya and Huang, 2018; Batabyal and Yeh, 2007; Kristeleit et al., 2017). Despite these early signals, the pivotal phase III ECHO-301 study combining epacadostat with pembrolizumab in advanced melanoma failed to demonstrate improved efficacy compared to pembrolizumab alone, leading to discontinuation of several ongoing programs (Long et al., 2019). This outcome highlighted crucial gaps in biomarker-driven patient selection, as well as the need to understand tumor microenvironment heterogeneity and IDO1 expression levels in order to enhance future trial designs.

### 5.10 IO102-IO103 (10)

IO102-IO103 **(10**, Figure 2) is a novel peptide vaccine developed by IO Biotech targeting IDO1 and PD-L1. It aims to stimulate T cell responses against immunosuppressive cells, enhancing anti-tumor immunity (IO Biotech, 2024). In phase 1/2 studies in combination with nivolumab in metastatic melanoma, IO102-IO103 showed an overall response rate (ORR) of 80% and a median progression-free survival of 26 months (Liu et al., 2010). Ongoing phase 2 trials are investigating its use with pembrolizumab in metastatic NSCLC, head and neck cancer, and urothelial bladder cancer (Kjeldsen et al., 2021; Tucker, 2023). The method of employing a vaccination strategy to educate and activate the immune system offers an alternative to direct enzyme inhibition, potentially extending therapy choices for patients who are less susceptible to checkpoint blockade alone.

### 5.11 M4112 (11)

M4112 **(11,** Figure 2 **)** is an orally administered dual inhibitor of IDO1 and TDO, developed by Merck Serono SA. It was the first dual IDO1/TDO inhibitor evaluated clinically, designed to address compensatory upregulation of TDO seen with selective IDO1 inhibition (Merck Serono SA, 2015). Although phase I trials showed acceptable safety, it did not reduce plasma kynurenine levels effectively, leading to early termination of the study (Naing et al., 2020). This emphasizes the difficulties in attaining adequate systemic suppression of the kynurenine pathway, as well as the necessity for improved pharmacodynamic indicators and optimal dual inhibitor designs.

### 5.12 LPM3480226 (12)

LPM3480226 (**12**, Figure 2, LY-01013) is a potent, selective dual inhibitor of IDO1 and TDO, developed by Luye Pharma Group Ltd. It targets the initial rate-limiting step in tryptophan metabolism to disrupt tumor-induced immunosuppression (Delving into the Latest Updatesb). It is currently being evaluated in an ongoing phase I trial for advanced solid tumors, with preliminary data suggesting acceptable safety and encouraging tolerability (Luye Pharma Group Ltd, 2025; ClinicalTrials, 2019a). More research is needed to establish its pharmacokinetic profile, effective pathway suppression, and patient selection criteria to enhance therapeutic benefit.

### 5.13 DN1406131 (13)

DN1406131 (**13**, Figure 2) is an orally active small-molecule inhibitor that targets both IDO1 and TDO2. By suppressing both IDO1 and TDO2, DN1406131 **13** attempts to prevent tryptophan depletion and kynurenine buildup within the tumor microenvironment, reactivating anti-tumor immunity (Opitz et al., 2020). The compound entered Phase I clinical testing (NCT03641794) to assess pharmacokinetics/pharmacodynamics in healthy volunteers and patients with advanced solid tumors, including glioblastoma, non-small cell lung carcinoma, and melanoma (Indoleamine 2,3-Dioxygenase (IDO) Inhibitor in Healthy Volunteers). Preclinical studies reveal that DN1406131 efficiently lowers kynurenine levels while increasing T-cell-mediated an. ti-tumor activity, especially when combined with immune checkpoint inhibitors or chemotherapy. Although additional clinical results have yet to be revealed, deliberate targeting of both IDO1 and TDO2 represents a promising method for overcoming immune escape mechanisms in a variety of solid cancers (Peng et al., 2022).

## 6 The safety profile and toxicity of multiple IDO/TDO inhibitors for cancer

The safety profiles summarized here include both monotherapies and various combination regimens (e.g., with chemotherapy or immune checkpoint inhibitors), as detailed in Table 2 for direct cross-reference and comparison. Overall, IDO and TDO inhibitors have shown acceptable tolerability, with most treatment-related adverse events (TRAEs) being grade 1–2. However, each agent demonstrates distinct safety considerations that warrant individual discussion below.

**TABLE 2:** Safety profile of IDO inhibitors in clinical trial (Phase 1, 2, 3).

Inhibitor name	Phase	Clinical Trial (Status and Disease)	Number of patients	Grade 1/2 AEs	Grade ≥3 AEs	Treatment-related death	Overall assessment
IDO inhibitors							
Epacadostat							
Epacadostat (INCB024360) monotherapy	1	**ID**: NCT01195311 **Status:** Completed in July 2013 **Disease**: Advanced solid tumors	52	**TRAEs** Fatigue (69.2%) Nausea (65.4%) ↓ appetite (53.8%) Vomiting (42.3%)	**TRAEs** Fatigue (11.5%) Nausea (9.6%) Vomiting (5.8%) **DLTs** Fatigue (2%) Radiation pneumonitis (2%)	0	Well- tolerated
Epacadostat + Pembrolizumab	1/2	**ID:** NCT02178722 **Status:** Completed in February 2021 **Disease**: Advanced solid tumors	62	**TRAEs (60%)** Fatigue (34%) Nausea (21%) Vomiting (10%) Diarrhea (18%) Pruritis (23%) ALT ↑ (7%) Arthralgia (22%) AST ↑ (8%) Rash (34%) **irAEs** Pneumonitis (2%) Colitis (2%)	**TRAEs (24%)** Fatigue (2%) Arthralgia (2%) AST ↑ (2%) Rash (8%)	0	Well- tolerated
Epacadostat + Pembrolizumab + 7 distinct chemotherapy regimens	1/2	**ID:** NCT03085914 **Status:** Completed in July 2020 **Disease:** Advanced solid tumors	70	**TRAEs (95.7%)** Fatigue (54.3%) Nausea (57.1%) Vomiting (32.9%) Diarrhea (41.4%) ALT ↑ (22.9%) AST ↑ (20%) **Hematological irAEs** Anaemia (40%) Thrombocytopenia (22.9%) Neutropenia (11.4%) Leukopenia (20%)	**DLTs (7.1%)** **Hematological irAEs** Neutropenia (24.3%)	0	Well- tolerated
Epacadostat + Pembrolizumab	2	**ID:** NCT03414229 **Status:** Active, not recruiting **Disease:** advanced sarcoma	30	**TRAEs** Fatigue (33.3%) Nausea (13.3%) Vomiting (6.7%), Diarrhea (3.3%) Pruritis (3.3%) Headache (6.7%) Fever (6.7%) ALT ↑ (10%) Arthralgia (16.7%) AST ↑ (13.3%) Rash (3.3%) **irAEs** Pneumonitis (6.7%) **Hematological irAEs** Anaemia (16.7%) Thrombocytopenia (3.3%)	**TRAEs** AST ↑ (6.7%) **Hematological irAEs** Anaemia (3.3%)	0	Well- tolerated
Epacadostat + ipilimumab	1/2	**ID:** NCT01604889 **Status:** Terminated in December 2016 **Disease:** Metastatic melanoma	50	**TEAEs** Fatigue (64%) Nausea (32%) Vomiting (24%) Diarrhea (34%) Headache (26%) ALT ↑ (28%) AST ↑ (24%) Rash (52%) Constipation (46%) Pruritus (38%) ↓ Appetite (26%) Cough (22%) Arthralgia (20%) **irAEs (80%)** Pruritis (28%) Rash (50%) ALT ↑ (28%) AST ↑ (24%) Hypothyroidism (10%)	**TEAEs (66%)** Fatigue (6%) ALT ↑ (16%) AST ↑ (16%) Anemia (2%) Confusional state (2%) Hyperglycemia (2%) Hyponatremia (2%) Hypotension (2%) Pruritus (2%) Urinary tract infection (2%) **irAEs (28%)** Pruritis (4%) Colitis (8%) ALT ↑ (16%) AST ↑ (16%)	0	Well- tolerated
Epacadostat + atezolizumab	1	**ID:** NCT02298153 **Status:** Terminated in November 2017 **Disease:** previously treated NSCLC	29	**TRAEs (79%)** Fatigue (38%) Decreased appetite (17%) Rash (17%) Nausea (17%) **irAEs (3.3%)** Throat tightness (3.3%)	**DLTs(7%)** **TRAEs (24%)** Hypotension (7%) Lipase ↑ (7%) **irAEs (10%)** Maculopapular rash (3.3%) Confusional state (3.3%) Autoimmune encephalitis (3.3%)	0	Well- tolerated
Epacadostat + durvalumab	1/2	**ID**: NCT02318277 **Status:** Completed in October 2020 **Disease:** advanced solid tumors	34	**TRAEs (79.4%)** Fatigue (32.4%) Nausea (11.8%) Diarrhea (11.8%) Headache (2.9%) Dyspnea (5.9%) AST ↑ (2.9%) **irAEs** Pruritis (17.6%) Maculopapular rash (5.9%)	**TRAEs (20.6%)** Fatigue (8.8%) Dyspnea (2.9%) **irAEs** Maculopapular rash (5.9%)	0	Well- tolerated
Epacadostat + durvalumab	1/2	**ID**: NCT02318277 **Status:** Completed in October 2020 **Disease:** advanced solid tumors	142	**TRAEs (80.3%)** Fatigue (30.3%) Nausea (23.2%) Vomiting (9.2%) Diarrhea (9.9%) Headache (6.3%) ALT ↑ (5.6%), AST ↑ (7.7%) ALP ↑ (6.3%) Rash (7.7%) **irAEs** Pruritis (11.3%) Maculopapular rash (12%)	**TRAEs (18.3%)** Fatigue (2.8%) Nausea (0.7%) Vomiting (1.4%) ALT ↑ (1.4%) AST ↑ (1.4%) ALP ↑ (1.4%) **irAEs** Maculopapular rash (4.2%)	0	Well- tolerated
Epacadostat + Pembrolizumab	3	**ID**: NCT02752074 **Status**: Completed in August 2019 **Disease:** advanced melanoma	354	**TRAEs (79%)** **irAEs** Pneumonitis (2%) Colitis (1%) Hepatitis (1%) Hyperthyroidism (6%) Hypothyroidism (11%)	**TRAEs (22%)** Lipase ↑ (4%) **irAE** Pneumonitis (1%) Colitis (1%) hepatitis (1%) Rash (1.1%)	0	Well- tolerated
Placebo + Pembrolizumab	3	**ID**: NCT02752074 **Status**: Completed in August 2019 **Disease:** advanced melanoma	352	**TRAEs (81%)** **irAEs** Pneumonitis (2%) Colitis (2%) Hyperthyroidism (7%) Hypothyroidism (9%)	**TRAEs (17%)** Lipase ↑ (3%) **irAEs** Pneumonitis (1%) Colitis (2%) Rash (2%)	0	Well- tolerated
Indoximod							
Indoximod monotherapy	1	**ID:** NCT00567931 **Status:** Completed in September 2012 **Disease:** Advanced solid tumors	48	**TRAEs** Fatigue (56.3) Anemia (37.5%) Anorexia (37.5%) Dyspnea (35.4%) Cough (33.3%) Nausea (29.2%) Hyperglycemia (22.9%) Vomiting (12.5%)	**TRAEs** Fatigue (4.1%) Anemia (6%) Dyspnea (6%) Hyperglycemia (2%)	0	Well- tolerated
Indoximod + docetaxel	1	**ID:** NCT01191216 **Status:** Completed in August 2013 **Disease:** Advanced solid tumors	27	**TRAEs** Fatigue (90%), Anemia (69%) Nausea (34%) Hyperglycemia (66%) Vomiting (28%) Diarrhea (28%) Constipation (28%)	**DLTs** Dehydration (7%) Colitis (3%) Hypotension (10%) Mucositis (3%**)** **TRAEs** Anemia (3%) Nausea (3%) Hyperglycemia (3%) Febrile neutropenia (13%) Neutropenia (13%) Constipation (28%)	0	Well- tolerated
Indoximod + temozolomide	1	**ID:** NCT02502708 **Status:** Completed in February 2020 **Disease:** pediatric brain tumors	54	**Any Events (94%)** Headache (43%) Vomiting (39%) Nausea (37%) Alopecia (13%) Diarrhea (15%) Constipation (19%) ALT ↑ (11%) Seizure (11%) Platelet count ↓ (9%)	**Any Events-Grade 3 (76%)** Headache (7%) Vomiting (15%) Nausea (4%) Diarrhea (2%) AST ↑ (2%) Seizure (7%) Platelet count ↓ (9%) **Any Events-Grade 4 (43%)** seizure (2%) Platelet count ↓ (26%)	0	Well- tolerated
Indoximod + taxane	2	**ID:** NCT01792050 **Status:** Terminated in June 2017 because of lack of efficacy. **Disease:** ERBB2-negative metastatic breast cancer	85	**TEAEs (68.2%)** Nausea (47.1%) Vomiting (23.5%) Diarrhea (35.3%) Fatigue (61.2%) Cough (20%) Alopecia (44.7%) Anemia (32.9%) Lymphopenia (23.5%) Hyperglycemia (23.5%) Extremities pain (15.3%)	**TEAEs (60%)** Nausea (2.4%) Vomiting (3.5%) Diarrhea (1.2%) fatigue (7.1%) anemia (8.2%) lymphopenia (3.5%) hyperglycemia (3.5%) extremities pain (1.2%)	0	Well- tolerated
Placebo + taxane	2	**ID:** NCT01792050 **Status:** Terminated in June 2017 because of lack of efficacy. **Disease:** ERBB2-negative metastatic breast cancer	79	**TEAEs (79.7%)** Nausea (48.1%) vomiting (35.4%) diarrhea (39.2%) fatigue (45.6%) cough (12.7%) alopecia (64.6%) anemia (19%) lymphopenia (12.7%) hyperglycemia (8.9%) extremities pain (10.1%)	**TEAEs (60.8%)** Nausea (2.5%) vomiting (1.3%) diarrhea (7.6%) fatigue (5.1%) Anemia (3.8%) lymphopenia (3.8%)	0	Well- tolerated
Indoximod + pembrolizumab	1/2	**ID:** NCT02073123 **Status:** Completed in July 2019 **Disease:** advanced melanoma	114	**TRAEs** Fatigue (62.3%) Nausea (28.1%) Vomiting (10.5%) Diarrhea (22.8%) Pruritis (35.1%) Arthralgia (21.9%) Rash (40.4%) ↓ appetite (18.4%) headache (18.4%) constipation (16.7%) hypothyroidism (13.2%) cough (11.4%)	**TRAEs** Fatigue (1.8%) Nausea (0.9%), Arthralgia (0.9%) Rash (4.3%) cough (0.9%)	0	Well- tolerated
Navoximod (GDC-0919)							
Navoximod monotherapy	1	**Status:** Phase I, completed **Disease:** Advanced solid tumors	10	**TRAEs (60%)** Chromaturia (50%) Maculopapular rash (20%)	**TRAEs (20%)** Maculopapular rash (10%) Lipase ↑ (10%)	0	Well- tolerated
Navoximod + atezolizumab	1	**Status:** Phase I, completed **Disease:** Advanced solid tumors	10	**TRAEs (100%)** Fatigue (20%) chromaturia (60%) ↓ appetite (30%) Hyponatremia (20%) AST ↑ (20%) ALT ↑ (20%)	**TRAEs (30%)** Hyponatremia (20%) AST ↑ (10%) ALT ↑ (10%)	0	Well- tolerated
Linrodostat (BMS-986205)							
Linrodostat + nivolumab	1/2	**ID:** NCT02658890 **Status:** Completed in February 2021 **Disease:** Advanced solid tumors	150	**TRAEs** fatigue (34%) nausea (21%) vomiting (10%) diarrhea (18%) pruritus (23%) ALT ↑ (7%) AST ↑ (8%) arthralgia (22%) rash (34%)	**TRAEs** Fatigue (2%) Arthralgia (2%) AST ↑ (2%) Rash (8%)	0	Well-tolerated
LY3381916							
LY3381916 Monotherapy	1a	**ID:** NCT03343613 **Status:** Terminated in May 2020 **Disease:** Advanced solid tumors	21	**TRAEs (67%)** Nausea (29%) fatigue (24%) vomiting (19%) AST ↑ (14%) ALT ↑ (10%) **SAE related to treatment (5%)**	**TRAEs (14%)** lipase increase (5%) maculopapular rash (5%) cholestasis (5%) **DLTs at 240 mg BID n = 1** ALT ↑ (5%) AST ↑ (5%) systemic inflammatory response syndrome (5%)	0	Well- tolerated
LY3381916 + LY3300054	1a	**ID:** NCT03343613 **Status:** Terminated in May 2020 **Disease:** Advanced solid tumors	21	**TRAEs (67%)** Nausea (24%) fatigue (24%) vomiting (5%) AST ↑ (10%) ALT ↑ (14%) **SAE related to treatment (10%)**	**TRAEs 29%** **DLTs at 240 QD n = 2** Fatigue (5%) Immune related hepatitis (5%)	0	Well- tolerated
LY3381916 + LY3300054 (TNCB Patients)	1b	**ID:** NCT03343613 **Status:** Terminated in May 2020 **Disease:** Advanced solid tumors	14	**TREAs (71%)** Nausea (21%) Vomiting (7%) ↓ appetite (29%) fatigue (29%) mouth (29%) AST ↑ (21%) ALT ↑ (7%) **SAE related to treatment** immune-mediated hepatitis (14%)	**TREAs (29%)** Lipase ↑ (7%) ALT ↑ (7%) neutropenia (7%) leukopenia (7%)	0	Liver toxicity
LY3381916 + LY3300054 (NSCLC Patients)	1b	**ID:** NCT03343613 **Status:** Terminated in May 2020 **Disease:** Advanced solid tumors	4	**TREAs** blurred vision (25%) ↓ appetite (25%) dizziness (25%) pruritus (25%)	**No TREAs/SAE (0%)**	0	Well- tolerated
KHK2455							
KHK2455 Plus Mogamulizumab	1	**ID:** NCT02867007 **Status:** Completed in December 2019 **Disease:** Advanced solid tumors	36	**TEAEs (100%)** Fatigue (33.3%) anemia (25%) hyperglycemia (11.1%) Vomiting (30.6%) diarrhea (22.2%) constipation (11.1%) ALT ↑ (22.2%) AST ↑ (16.7%) lymphopenia (22.2%) dyspnea (22.2%) **Serious TEAEs (38.9%)** Nausea (38.9%) drug eruption (55.6%)	**TEAEs (61.1%)** **DLTs** Gastrointestinal necrosis (2.8%)	0	Well- tolerated
PF-06840003							
PF-06840003 monotherapy	1	**ID**: NCT02764151 **Status**: Completed in December 2018 **Disease:** recurrent malignant glioma	17	**TEAEs (100%)** Fatigue (52.9%) anemia (52.9%) hyperglycemia (29.4%) diarrhea (11.8%) headache (29.4%) **TRAEs (82.3%)** Fatigue (47.1%) anemia (35.3%) hyperglycemia (11.8%) Vomiting (17.6%) AST ↑ (5.8%) Ejection fraction ↓ (5.8%)	**TRAEs** ALT ↑ (11.8%) AST ↑ (5.8%) Ejection fraction ↓ (5.8%) DLTs (12.5%)*	0	Well- tolerated
NLG-802 (prodrug of indoximod)							
NLG-802	1	**ID**: NCT03164603 **Status**: Completed in 2019 **Disease**: Advanced solid tumors	24	nausea (33%) fatigue (25%) diarrhea (20%) ↓ appetite (17%)	↑ liver enzyme (4%)	0	Well-tolerated
LPM-3480226							
LPM-3480226 (LY-01013)	1	**Status:** Active, not recruiting **Disease**: Advanced solid tumors (early-stage cohort)	Not fully disclosed yet (early enrollment ongoing)	**Preliminary reports**: Fatigue Nausea Diarrhea No severe AEs reported yet	Not yet reported in detail	0	Preliminary data suggest acceptable safety
Dual Inhibitors (IDO and TDO)							
HTI-1090							
HTI-1090 (SHR9146)	1	**ID**: NCT03491631 **Status**: Completed in 2021 **Disease**: Advanced solid tumor	23	Fatigue (35%) ↓ appetite (30%) rash (15%) nausea (10%)	↑ liver enzyme (5%) hypertension (4%)	0	Well tolerated
M4112							
M4112 monotherapy	1	**ID:** NCT03306420 **Status:** Completed in December 2018 **Disease:** Advanced solid tumors	15	**TEAEs (100%)** Fatigue (33.3%) nausea (26.7%) Vomiting (26.7% AST ↑ (13.3%) ALT ↑ (13.3%) lipase ↑ (20%) **Serious TEAEs (20%)**	**TEAEs (40%)** Diarrhea (6.6%) Rash (6.6%) DLTs (6.6%)** Allergic dermatitis (6.6%)	0	Well- tolerated
DN1406131							
DN1406131	1	**Status**: Active, recruiting **Disease**: Advanced solid tumors (early-stage, first-in-human)	Not fully disclosed yet (early enrollment ongoing)	fatigue nausea ↓ appetite vomiting reported no detailed breakdown yet	Not yet reported	0	Preliminary data suggest acceptable safety profile

Abbreviations: AEs, adverse events; TRAEs, treatment-related adverse events; TEAEs, treatment-emergent adverse events; irAEs, immune-related adverse events; DLTs, dose-limiting toxicities; DLTs*, dose-limiting toxicity at dose 500 mg, BID (N = 1/8); DLTs**, dose-limiting toxicity at dose 800 mg BID (N = 1/15); ALT, alanine aminotransferase; AST, aspartate aminotransferase; ALP, alkaline phosphatase; SAE, serious adverse event; NSCLC, non-small cell lung cancer; TNCB, triple-negative breast cancer; QD, once daily; BID, twice daily; ↑ = increase; ↓ = decrease.

### 6.1 Epacadostat

In phase I and II studies, epacadostat generally showed an acceptable safety profile alone or combined with immunomodulatory agents such as pembrolizumab, ipilimumab, or atezolizumab. When used alone, TRAEs were well tolerated up to 700 mg BID. In combination, doses up to 100 mg BID were safe and tolerable, except when combined with ipilimumab, where ≤50 mg BID was acceptable (Beatty et al.).

The phase III study combining epacadostat and pembrolizumab demonstrated a safety profile similar to pembrolizumab plus placebo, with no TRAEs leading to death (Mitchell et al., 2018). Common adverse events included fatigue, rash, pruritus, and elevations in liver enzymes. More research is needed to determine its pharmacokinetic profile, effective pathway suppression, and patient selection criteria for maximum therapeutic effectiveness.

### 6.2 Indoximod

Indoximod has shown favorable safety and activity when used with chemotherapy across different types of cancer. The recommended phase II dose is 1,200 mg orally twice daily (Soliman et al., 2014; Soliman et al., 2016). In multiple studies, including combinations with docetaxel or pembrolizumab, indoximod was generally well tolerated, with most adverse events being grade 1/2 (Johnson et al., 2024; Mariotti et al., 2021). Notably, unlike other IDO inhibitors, indoximod showed no substantial hepatotoxicity signal, indicating that it should be studied further in combination regimens (Zakharia et al., 2021).

### 6.3 Navoximod

Navoximod demonstrated acceptable tolerability as monotherapy and in combination with atezolizumab, with no grade 4/5 TRAEs reported. In Japanese cohorts, most adverse events were grade 1/2, including fatigue and nausea, with few grade 3 events. Future development should focus on efficacy optimization through patient selection and combination strategies (Ebata et al., 2020).

### 6.4 PF-06840003

PF-06840003 was assessed in recurrent malignant glioma but was discontinued early due to strategic reasons despite demonstrating acceptable safety at lower doses. In a phase I study of patients with recurrent malignant glioma, PF-06840003 was generally well tolerated up to 500 mg BID. No treatment-related deaths were reported, though liver enzyme elevations (AST and ALT) were noted at higher doses (Reardon et al., 2020).

### 6.5 KHK2455

KHK2455 combined with mogamulizumab showed an acceptable safety profile in phase 1 clinical trials, with most patients experiencing grade 1–3 treatment-emergent adverse events (TEAEs), primarily fatigue, diarrhea, and rash. Only one dose-limiting toxicity was reported, and no treatment-related deaths occurred (Sahebjam et al., 2020b)**.**

### 6.6 LY3381916

LY3381916 was tolerable as monotherapy up to 240 mg daily. However, in combination with PD-L1 inhibitors in triple-negative breast cancer, high rates of grade 3 liver toxicity (35.7%) were observed, leading to early termination of some study arms (Kotecki et al., 2021).

### 6.7 HTI-1090

HTI-1090 (SHR9146) has shown favorable safety in early-phase studies, with most adverse events being grade 1–2 (e.g., fatigue, decreased appetite, rash). No treatment-related deaths have been reported, and it remains a promising dual IDO/TDO inhibitor under further evaluation.

### 6.8 M4112

M4112 was generally well tolerated in early-phase studies, with no major safety concerns. However, pharmacodynamic failure to adequately suppress kynurenine limited clinical progress and led to early study discontinuation (Naing et al., 2020).

### 6.9 LPM3480226

LPM3480226 has shown preliminary tolerability in ongoing phase I trials. Further data from ongoing phase I trials are awaited to fully establish its safety and potential combination strategies (ClinicalTrials, 2019a).

### 6.10 DN1406131

DN1406131 is a novel dual IDO/TDO inhibitor currently in first-in-human phase I trials. Preliminary reports suggest acceptable safety, with commonly reported adverse events including fatigue, nausea, decreased appetite, and vomiting. No severe adverse events or treatment-related deaths have been reported to date.

### 6.11 NLG802

NLG802, an oral prodrug of indoximod, has shown good tolerability in early-phase trials, with commonly reported adverse events including nausea (33%), fatigue (25%), diarrhea (20%), and decreased appetite (17%). No treatment-related deaths were reported, supporting its further investigation as an improved delivery form of indoximod (NewLink et al., 2020; Mautino et al., 2017).

### 6.12 Linrodostat (BMS-986205)

Linrodostat, used alone or with nivolumab, was generally well tolerated, with common adverse events including fatigue, nausea, vomiting, diarrhea, pruritus, and liver enzyme elevations. Grade 3 or higher TRAEs were relatively infrequent. No treatment-related deaths were reported, and the combination showed promising safety in solid tumors (SITC, 2017; Balog et al., 2021; Luke et al., 2019).

## 7 Effective IDO/TDO drug candidates in cancer treatments

### 7.1 IDO1 and colorectal cancer (CRC)

Colorectal cancer (CRC) is the second leading cause of cancer death and the third most commonly diagnosed cancer globally (Peng et al., 2022). High IDO1 expression in CRC is associated with immunological tolerance, metastasis, and poor prognosis. The serum Kyn/Trp ratio has been proposed as a screening and prognostic marker (Sung et al., 2021). Kynurenine acts as an AhR agonist, inducing Treg development, PD-1 expression in CD8^+^ T cells, and accelerating carcinogenesis in the intestinal epithelium. IDO and Kyn pathway metabolites stimulate β-catenin and PI3K-Akt pathways, enhancing proliferation and inhibiting apoptosis (Siegel et al., 2020). Interestingly, while IDO1’s role in immune suppression is established in other cancers, the exact mechanisms in CRC remain unclear, with potential contributions from tumor-associated microbiota, such as *Fusobacterium* nucleatum (Ala, 2021; Han et al., 2021; Bishnupuri et al., 2019). This bacterium can promote tumor immune evasion and may modulate IDO1 expression *via* inflammatory macrophage interactions (Xue et al., 2018).

### 7.2 IDO1 and breast cancer

Breast cancer (BC) remains the most common cancer among women worldwide and is responsible for the highest female cancer mortality (Wilkinson and Gathani, 2022; Luo et al., 2018). IDO1 is upregulated in BC, promoting Treg infiltration, metastasis, and immune suppression (Yu et al., 2011). L-Kyn induces ROS generation, leading to NK cell apoptosis and facilitating tumor evasion (Song et al., 2011). IDO1-mediated MDSC activity is STAT3 dependent, contributing to immune suppression and angiogenesis (Yu et al., 2013; Wei et al., 2018).

Furthermore, IDO1 expression is inversely related to estrogen receptor α (ERα) expression. Studies suggest that ER-positive tumors show reduced IDO1 levels due to promoter methylation mechanisms facilitated by ERα (Dewi et al., 2017). This interplay highlights the complexity of immune evasion and potential for combinatorial strategies targeting IDO1 and hormone signaling pathways.

### 7.3 IDO1 and melanoma

IDO1 overexpression in melanoma aids immune evasion, correlates with Breslow thickness, and predicts poor prognosis (Lynch et al., 2021; Weinlich et al., 2007). Melanoma patients frequently display elevated Kyn/Trp ratios, indicating systemic immunosuppression, which can be reversed with IDO1 inhibition (Slingluff et al., 2018). IDO1 expression is associated with peritumoral inflammatory infiltrates and tumor aggressiveness, emphasizing its potential as a therapeutic and prognostic biomarker.

### 7.4 IDO and gastric cancer

Gastric cancer ranks third globally in cancer mortality. IDO1 expression correlates with lymphocyte exclusion, poor survival, and chemoresistance (Lian et al., 2012; Nishi et al., 2018; Kim et al., 2016). Tryptophanyl-tRNA synthetase (WARS) facilitates high-affinity Trp uptake, supporting tumor growth and further reducing local Trp availability. Moreover, IDO1 expression is associated with TGF-β signaling and increased Foxp3+ Treg infiltration, indicating a highly immunosuppressive tumor microenvironment (Miyanokoshi et al., 2018).

### 7.5 IDO and pancreatic cancer

IDO1 activity in pancreatic cancer supports profound immunosuppression by promoting Trp catabolism and Kyn production (Liang et al., 2021), IDO1 also contributes to one-carbon metabolism, providing folate-dependent nucleotides, thereby linking immune evasion with metabolic flexibility. Tryptophan was used as a one-carbon donor in place of serine, and serine restriction enhanced the anti-tumor efficacy of the IDO1 inhibitor epacadostat. High IDO1 expression is associated with advanced disease and poor prognosis, while lower levels correlate with better survival outcome (Newman et al., 2021).

### 7.6 IDO1 and endometrial carcinomas

Endometrial carcinoma (EC) is the most common gynecological malignancy in Europe. High IDO1 expression is linked to Treg infiltration and immune evasion, correlating with poor prognosis. Frequent co-expression of PD-L1 and IDO1 suggests a cooperative mechanism in immune suppression and a potential dual target for immunotherapy (Tumeh et al., 2014; Sloan et al., 2017; Liu et al., 2015; Mills et al., 2018).

### 7.7 IDO1 and esophageal cancer (EC)

Esophageal carcinoma shows elevated IDO1 expression, which promotes tumor cell proliferation and migration *via* NF-κB–CXCL10 signaling (Harada et al., 2020; Yao et al., 2023). IDO1 and PD-L1 co-expression is associated with high T cell infiltration but paradoxically worse survival, reflecting complex immune evasion mechanisms (Dufour et al., 2002; Tokunaga et al., 2018). Stromal fibroblasts and capillaries in esophageal squamous cell carcinoma have been shown to drive IDO1 expression, sustaining a tolerogenic microenvironment (Rosenberg et al., 2018; Cui et al., 2018).

### 7.8 IDO1 and thyroid carcinoma

Thyroid carcinoma exhibits high IDO1 expression, driven by BRAF mutations and RET/PTC rearrangements (La Vecchia et al., 2015). IDO1-mediated kynurenine production suppresses lymphocyte proliferation, fostering an immune-privileged niche and facilitating tumor progression. *In vitro*, thyroid cancer cell lines secrete high levels of Kyn, directly suppressing co-cultured lymphocyte proliferation (Moretti et al., 2014).

Collectively, these findings highlight IDO1 as a multifaceted therapeutic target across diverse malignancies, warranting further clinical exploration of selective and combination approaches.

## 8 Challenges and future directions

Despite promising preclinical and early clinical outcomes, various difficulties have hampered the use of IDO and TDO inhibitors in clinical practice. A key impediment has been the lack of strong predictive indicators to identify individuals who would benefit the most from these treatments. Despite encouraging previous studies, Epacadostat failed in the Phase III ECHO-301 study, emphasizing the significance of patient classification and a full understanding of the tumor microenvironment.

Tumor heterogeneity, both within and between tumours, exacerbates treatment results. IDO and TDO expression levels can vary greatly between cancer types and among tumor subregions. The dynamic and immunosuppressive tumor microenvironment, driven by nutritional depletion, cytokine gradients, and metabolic interactions, has a substantial impact on the efficacy of these inhibitors. Furthermore, recent research indicates that microbiome-derived metabolites may influence tryptophan metabolism and immunological responses, adding another degree of complexity.

Furthermore, the functional redundancy and potential compensation between IDO1 and TDO2 strengthen the case for dual inhibition techniques. While most previous attempts have focused on IDO1, data suggests that TDO2 can compensate for IDO1 inhibition, promoting the development of dual inhibitors such as HTI-1090 and M4112.

Future research should focus on incorporating comprehensive biomarker studies, such as metabolic signatures, immunological profiling, and microbiome assessments, into clinical trial designs. Combination therapies with immune checkpoint inhibitors, chemotherapy, or radiotherapy are promising techniques for increasing efficacy and overcoming adaptive resistance. Systems biology and computational modeling approaches may potentially be useful in guiding individualized treatment options and optimizing medication combinations. Addressing these difficulties is critical to realizing IDO and TDO inhibitors’ full potential as breakthrough cancer immunotherapy drugs.

Furthermore, more investigation of IDO/TDO expression heterogeneity across different tumor types and microenvironments is required to improve patient selection and response prediction. Integrating knowledge of microbial interactions with tryptophan metabolism could help explain individual variability in treatment outcomes. Furthermore, using systems biology and computational modeling could provide comprehensive insights into pathway dynamics and reveal novel vulnerabilities for targeted therapy. Thus, a multifaceted strategy combining molecular insights, precision medicine tools, and innovative trial designs will be key to overcoming current barriers.

## 9 Conclusion

IDO and TDO inhibitors offer a compelling strategy to overcome tumor-induced immunosuppression and enhance antitumor immunity. Agents like Indoximod have demonstrated encouraging clinical safety profiles and the ability to enhance T cell activation, while Epacadostat and other IDO1-selective inhibitors showed promise but highlighted the importance of patient selection and combination strategies. Dual inhibitors, such as HTI-1090, present a potential approach to address redundancy in the kynurenine pathway, yet they face challenges in achieving effective systemic suppression without off-target metabolic effects. Future research should focus on refining biomarker-driven patient stratification, integrating microbiome interactions, and optimizing combination regimens with immune checkpoint inhibitors or targeted therapies. Additionally, exploring the tumor microenvironment heterogeneity, addressing tumor-specific metabolic dependencies, and minimizing systemic immune-related adverse events will be crucial for maximizing therapeutic success. As these avenues are further developed, IDO and TDO inhibitors are poised to reshape cancer immunotherapy paradigms, potentially transforming patient outcomes and broadening the scope of immunologically targetable tumors.
